# Novel polyclonal antibody-based rapid gold sandwich immunochromatographic strip for detecting the major royal jelly protein 1 (MRJP1) in honey

**DOI:** 10.1371/journal.pone.0212335

**Published:** 2019-02-19

**Authors:** Yifan Zhang, Yong Chen, Yiting Cai, Zongyan Cui, Jinjie Zhang, Xiaohou Wang, Lirong Shen

**Affiliations:** 1 Department of Food Science and Nutrition, Zhejiang Key Laboratory for Agro-Food Processing, Zhejiang University, Hangzhou, Zhejiang, China; 2 Qinhuangdao Entry–Exit Inspection and Quarantine Bureau, Qinhuangdao, Hebei, China; VIT University, INDIA

## Abstract

Honey adulteration is becoming increasingly alarming incidents in food safety. Monitoring and detecting adulteration face greater challenges. Honey contains the major royal jelly proteins (MRJP) secreted by bee workers. To detect honey adulteration fast and accurately, a rapid gold sandwich immunochromatographic strip (GSIS) was developed based on two specific polyclonal antibodies (PoAbs) against the MRJP1, the most abundant protein of all MRJPs. We determined the best of pH value (pH 8.6) and PoAb SP-1 amount (5 μg/mL) in conjunction with colloidal. The cut-off value (sensitivity) of GSIS in detecting MRJP1 is 2.0 μg/mL in solution. Validation analysis with RJ, milk vetch honey, acacia honey and honey adulteration containing rice syrup and corn syrup with different ratios demonstrated that the GSIS could show consistent Test line (T line) when the test samples contain more than 30% pure honey or MRJP1 0.4 mg/g. The validation results by isotope ratio mass spectrometry on the same pure and all adulteration milk vetch honey samples showed the same information of GSIS test. The qualitative assay GSIS provided a valuable new way for honey authenticity and laid the foundation for the future application of GSIS with monoclonal antibodies in honey authentication.

## Introduction

Honey is produced by honeybees from the nectar of plants or honeydew secretions of plants combined with their own secretions. It is mainly composed of sugar, mainly fructose and glucose, organic acids, amino acids, proteins, minerals, and other phyto-chemicals [1]. In addition, the composition of honey depends on its geographical and botanical origins. Moreover, honey exhibit a wide range of nutritional and biological effects such as antibacterial property, antioxidant property, antimutagenic property, anti-inflammatory and healing property, etc [2]. As a nutrient-rich natural sweetener and healthcare product, honey is one of the global favored foodstuffs. However, honey is easily adulterated in the practice by cheaper, commercially available sugar syrups with similar composition because of its composition (mainly carbohydrates and water) [3]. Economically motivated adulteration of honey has widely affected global honey producers, retailers, consumers and regulatory authorities [2].

Many detection methods have been developed and used to identify adulteration of honey in recent decades. These include high-performance liquid chromatography (HPLC) [4], gas chromatography–mass spectrometry (GC-MS) [5], near-infrared (NIR) spectroscopy [6], Fourier transform infrared (FTIR) spectroscopy [7], electronic tongue [8], fluorescence spectroscopy [9], Raman spectroscopy [10], high-resolution nuclear magnetic resonance (HR-NMR) [11], and isotope ratio mass spectroscopy (IRMS) [12], pollen identification [13], stable carbon isotope ratio method [14], etc. However, detection methodology of adulteration lagging behind adulteration technology has made us to rethink of conventional strategy. Over the past 20 years, the advances in mass spectrometry, high flux antibodies, and bioinformatics and biostatistics algorithm biomarkers based on genomics, proteomics, and metabolomics have been developed and applied in detection of honey adulteration [15]. Biomarkers, such as DNA and proteins have been identified and used to determine adulteration and floral sources of honey [16–17].

Traditional methods of honey authenticity have failed for two main reasons. First, it takes time to develop new detection technology against adulteration when a new adulteration method is known. This situation creates a “window-blank period” for monitoring honey adulteration and results in an early warning and effective control of new adulterated technologies could not be provided in time. During this “window period”, the risk of food safety caused by honey adulteration to consumers will increase drastically. Secondly, the cost of monitoring and controlling adulteration is too high. To some extent, modern analytical techniques can effectively identify the authenticity of honey, which is beneficial to the quality control of honey [2, 8]. However, there were shortcomings such as expensive instruments and equipment, complex operation techniques, long testing time, professional background, and fixed operation sites for existing analysis technologies. At the same time, a single technology has limited detection range, which cannot guarantee the accuracy of the results. It is necessary to analyze the cross-examination of chemistry, organic chemistry, biology, pop analysis and other disciplines to determine the authenticity of honey accurately [3, 18]. As the existing identification and analysis technology lag behind the adulteration technology, it is difficult to eliminate honey adulteration completely. Therefore, the development of a new honey adulteration detection technology should change strategy from the past, to determine "if the sample contains adulteration" to "what the sample contains," The trend of authenticity test is increasingly recognized in the analytical methods of honey [19]. In fact, the ideal method of honey identification is accurate, fast, convenient and inexpensive, without professional technical training. In view of existing defects for honey identification technology, it is essential to change strategies from aiming exogenous substances added to the honey mainly to focusing on the honey authenticity unique composition from now on.

Honey contains 1–2% protein. Although major royal jelly proteins (MRJP) are found in the royal jelly (RJ) secreted from salivary glands of worker bees at high concentrations, it is also present in honey at a low concentration [20]. MRJP family is composed of nine members: MRJP1 to MRJP9. Mass spectrometric analysis showed that honey contained MRJP1, MRJP2, MRJP5, and MRJP7. MRJP1 is the most abundant protein in honey [1]. According to its stability and immune characteristics in honey, it is feasible to use MRJP1 as the honey endogenous biomarker for honey quality. In recent years, we have constructed a fast ELISA method to determine the RJ freshness with a specific antibody of MRJP1 [21–22], which provided the foundation to develop a new way to detect MRJP1 in honey. ELISA is a simple, specific, and cost-effective method to detect MRJP1, but it is time-consuming and unstable procedure [23].

The colloidal gold immunochromatographic strip (CGIS) assay is a rapid, reliable, easy, economical and instrument-free analytical method. CGIS has been widely used in detecting residues of pesticides and veterinary drugs, biological and chemical contaminants in food products, medical diagnosis, etc. In comparison with typical chromatography, CGIS has been confirmed as an alternative for measurement of detection target such pesticides by virtue of its high selectivity, sensitivity, and reliability as well as its speed, which enables detection visually within a few minutes [24]. Based on our previous ELISA method to detect RJ freshness [22], CGIS assay which determines the authenticity by detecting internal biomarker MRJP1 in honey was developed in this study.

## Materials and methods

### Materials

#### Chemical materials

Bovine serum albumin (BSA), polyvinyl pyrrolidone (PVP), polyethylene glycol 2000 (PEG 2000), and polyvinyl alcohol (PVA) were purchased from Aladdin Chemistry Co., Ltd. (Shanghai, China), and 3,3′,5,5′-tetramethylbenzidine (TMB), enzyme immunoassay grade horseradish peroxidase (HRP)-labeled goat anti-rabbit immunoglobulin were purchased from Beyotime Biotechnology Co., Ltd (Hangzhou, China). Other chemicals were from the National Pharmaceutical Group Chemical Reagent Co., Ltd. (Shanghai, China). All biochemical reagents were of highest purity and commercially available. Nitrocellulose (NC) membrane (pore size = 5–8 μm) was provided by Sartorius (Germany). Sample pad and adsorbent pad were the products of Millipore (USA). Semirigid polyvinyl chloride (PVC) sheets with adhesive tape were purchased from a local market. The reference standard, olive oil (%C = 57.00, δ^13^C_PDB_ = −29.07‰), which was re-certified by the National Research Center for Certified Reference Materials (China), copper oxide (0.7 mm), copper (0.7 mm reduced), chromium oxide (granular), magnesium perchlorate (granular), silver wool and quartz wool were purchased from PDZ Europa (Cheshire, UK). Reagent grade sulfuric acid and analytical grade sodium tungstate (Na_2_WO_4_·2H_2_O) were purchased from Beijing Chemical Ltd (China). The helium carrier gas, auxiliary combustion gas and oxygen were 99.999% pure.

Pure natural milk vetch honey (floral source: *Astragalus sinicus* L.) was provided by Nanchang Tongxin Zichao Biological Engineering Co., Ltd.(Nanchang, China. It was produced in December, 2014). Pure natural acacia honey (floral source: *Robinia pseudoacacia* Linn. It was produced in November, 2014) and fresh RJ were provided by Hangzhou Biyutian Health Food, Co., Ltd. (Hangzhou, China). All honey samples were stored at 4°C in refrigerator. Fresh RJ was stored at −80°C until use. MRJPs were extracted from fresh diluted in phosphate buffered saline (PBS) by centrifugation at 12,000×g for 30 min under 4 °C, then dialyzed against 500 volumes of ddH_2_O via distilled by using a 1000 Da cut-off dialysis membrane under 4°C [25]. The concentration of MRJPs was determined by Bradford using bovine serum albumin as standard. MRJP1 was prepared with ultracentrifugation method from RJ as previously described [26].

#### Buffer solutions

The following solutions were used: 0.05 M sodium carbonatebicarbonate buffer (CB, pH 9.6); 0.05 M sodium CB buffer containing 0.2% (w/v) gelatin as blocking buffer; 0.01 M PBS (pH 7.4); 0.01 M PBS containing 0.05% (v/v) Tween-20 (PBST, pH 7.4) as washing buffer; 0.01 M PBS containing 0.1% (w/v) gelatin as an antibody dilution buffer; 0.01 M PBS containing 5% (w/v) skim milk powder as blocking buffer; and 2 M H_2_SO_4_ [10%(w/v)] as stop reagent.

#### Preparation and purification of PoAbs against MRJP1

The oligopeptides I-SGEYDYKNNYPSDID (P1) and 2-IKEALPHVPIFD (P2) were selected by homology analysis of nine MRJP family sequences from GenBank web (http://www.ncbi.nlm.nih.gov) by using Genetyx-Win Version 5 (Software Development Co., Tokyo, Japan) [22], and synthesized using the AMS 422 robot (ABIMED GmbH, Langenfeld, Germany) by China Peptides Co., Ltd., respectively (Bradford, 1976). P1 and P2 were prepared into specific PoAbs, SP-1 and SP-2 against MRJP1. The sensitivity and specificity of SP-1 and SP-2 were measured by using indirect ELISA [22]. Then, by labeling with horseradish peroxides (HRP), SP-1 was prepared into detecting antibody, HRP-SP-1. SP-2 was used as the capture antibody in a newly established sandwich ELISA for honey.

#### Standard solutions preparation

The MRJP1 standard was dissolved in PBS (1 M, pH 7.4) to give the stock solution (1 mg/mL). Then the stock solution was serially diluted with PBS to give the working standard solutions.

### Titer determination of PoAbs against MRJP1

Indirect ELISA was used for determining the titer of both antibodies SP-1 and SP-2 according to the method of Anuracpreeda [27]. Briefly, a 96-well microtiter plate (Costar, America) was coated with 100 μl of 1 μg/ml of MRJP1 from RJ diluted in coating buffer, and incubated at 4°C overnight. The coated plate was washed three times with PBST, and the washing fluid was left in the wells for nearly 1 min each time. Afterward, the plate was blocked with 200 μl/well of blocking solution in 0.01 M PBS (pH 7.4) at 37°C for 1.5 h. Next the plate was washed with 0.01 M PBST; and 100 μl of diluted SP-1 or SP-2 at 1:1250, 1:2500, 1:5000, 1:10000, 1:20000, 1:40000 and 1:80000 were added and incubated at 37°C for 1 h, respectively. The blank rabbit serum (the control) was used as the negative control at 1:1000. After being washed with 0.01 M PBST, the plate was incubated with 100 μl/well of HRP-labeled goat anti-rabbit immunoglobulin, at 1: 250 dilution in the 0.01 M PBS (pH 7.4) at 37°C for 30 min. Then the plate was washed with PBST as previously described, and the color development was generated by attaching 200 μl/well of TMB substrate. The enzymatic reaction was released to take place at 37°C for 15 min in dark. Finally, the enzymatic reaction was stopped by addition of 50 μl stop reagent. The optical density (OD) value at 450 nm and 630 nm was measured using a Multiskan MK microplate reader (Thermo Fisher, Shanghai, China).

### Preparation and identification of colloidal gold nanoparticles (GNPs)

In the selection of colloidal gold particle size, 40 nm colloidal gold was the optimal particle size for most diagnostic applications, taking into account the balance between the required visibility and steric hindrance [28–29]. GNPs were synthesized with the sodium citrate reduction method [30]. Firstly, 1 mL 1% (w/v) HAuCl_4_·4H_2_O was added to 100 mL ultrapure water in a magnetic stirring reflux device, and heated to boiling. Then, the freshly prepared 1% (w/v) trisodium citrate solution (1 mL) was rapidly added. The solution color changed from colorless to wine red within 8 min. The resulting GNPs were stored at 4 °C and characterized by transmission electron microscopy and UV-vis after the mixture cooled to room temperature.

### Optimization of pH and PoAb SP-1 content for the sandwich immunoassay

The best binding force of colloidal gold and protein is obtained under the condition of protein isoelectric point (PI). The pH value of the colloidal gold solution was adjusted to 3, 4, 5, 6, 7, 8, 9, 10; 3 μL SP-1 (1 mg/mL) was added to 100 μL colloidal gold solution with different pH values, mixed in the plate. 20 μL 10% (w/v) NaCl was added after 15 min. The colloidal gold color change observed and the minimum pH (X) of the mixture for keeping the red was recorded within 10 min. The pH value of the gold solution was adjusted to X-0.6, X-0.3, X, X+0.3, X+0.6, X+1, respectively, then the above steps were repeated. The results were observed after incubation for 2 h. The amount of SP-1 protein in conjunction with colloidal gold was optimized as well. 0, 1, 2, 3, 4, 5, 6 μL SP-1 solutions (1 mg/mL) were added to a 1 mL centrifuge tube with colloidal gold solution (pH 8.6), respectively. After 15 min, 100 μL NaCl solution [10% (w/v)] was added to each tube and mixed well. The results were observed after 2 h.

### GNP-labeled PoAb

The GNP solution was adjusted to pH 8.6 with 0.1 M K_2_CO_3_ before PoAb was labeled. Then, 0.4 mg PoAb (5 μg/mL) in 0.01 M PBS at pH 8.6 was added drop-wise into 4 mL GNP solution and bred at room temperature for 1 h for conjugation. To block the gold nanoparticles, 4 mL 0.5% BSA (w/v) and 0.01% PEG 2000 (w/v) was slowly added into the solution to stabilize the labeled PoAb. After incubation for 1 h, the solution was centrifuged at 10000 r/min for 30 min, and the precipitate was dissolved with 0.01 M PBS (containing 1% BSA, and 0.01% PEG 2000, pH 8.6), then the solution was centrifuged at 10000 r/min for 30 min again. The precipitate was resuspended with 0.01 M PBS (containing 1% BSA, and 5% sucrose, pH 8.6) and stored under 4 °C after repeating the above operation twice, and finally [30].

### The compound of the immunochromatographic strip (IS)

The IS was assembled layer by layer with a sample pad, NC membrane, PVC sheet, and absorption pad. Different proteins were sprayed onto the NC membrane at 0.05 μL/mm by using a membrane dispenser machine (IsoFlow, USA), and dried under room temperature. SP-2 was sprayed on the test zone to capture object protein in the samples, and goat anti-rabbit IgG was sprayed on the control zone for quality control. These strips were sealed in plastic bags containing desiccant and stored in a clean desiccator under 4°C until required. The test strip consists of a plastic backing plate on which the NC membrane, sample pad, and absorbent pad are pasted. After assembly of each component, the whole assembled plate was cut into lengthways and divided into strips with a guillotine cutter (3×60 mm) [31].

### Application of strip detection

Before detection, 8 μL GNP-labeled SP-1 was added to the sample pad for testing. Then the strip pad has to be immersed in the test solution (100 μL) when detection. The strip surface must be lower than the sample pad, allowing the sample to migrate upward. After 10 min, the testing result could be checked.

### Handling of honey samples

100 g of each honey sample was diluted with pure water (1:1), mixed evenly and extracted overnight at 4°C. The supernatant was harvested after the honey solution was centrifuged at 12,000×g for 30 min under 4°C. Then the extract from honey water extract was dialyzed against 500 volumes of ddH_2_O via distilled by using a 1000 Da cut off dialysis membrane under 4°C.

### Detection of honey adulteration with strip detection

Milk vetch honey was mixed with corn syrup and rice syrup with following mass ratios, 10: 0, 7: 3, 5: 5, 3: 7, 0: 10, respectively. The MRJP1 contents in all milk vetch honey mixture and pure Acacia honey were detected by ELISA with SP1 as antibody against MRJP1 according to our previous report [22]. All mixtures were treated as above. 80–100 μL sample solution was added to each well of 96-well plate. Then, the strips were dipped into the sample solution and taken out for 8–10 min to observe the result. If both the test (T) and control (C) lines showed a red mark, the samples were recorded as positive indicating the presence of MRJP1. On the contrary, if the C line but not T line showed a red mark, the sample is considered as negative. The strip test was invalid when there was no red mark in the control region.

### Determination of honey adulteration by elementary analysis-isotope ratio mass spectrometry (EA-IRMS)

EA-IRMS determination was performed according to AOAC method 998.12 [32] and previous report [33] with a little modification. EA-IRMS (Integra-CN stable carbon and nitrogen isotope analyser) and tin capsules (8 mm × 5 mm) were purchased from PDZ Europa. For honey samples, 3 μL of original honey sample was pipetted into a tin capsule. For honey protein samples, 20–25 g of honey sample was mixed well with 5 mL of water in a 50 mL centrifuge tube. Then 2 mL of 100 g/L sodium tungstate solution and 2 mL of 0.335 mol/L sulfuric acid solution were mixed well in a 20 mL measuring cylinder, added to the centrifuge tube containing the honey sample and again mixed well. The centrifuge tube was subsequently placed in an 80 °C water bath for at least 30 min until a visible protein floc had formed, with the tube being swirled for 20 s at 5–10 min intervals during the heating process. Then 30 mL of water was mixed with the contents of the centrifuge tube, the tube was centrifuged for 5 min at 1500 × g and the supernatant was decanted. A further 50 mL of water was added to the centrifuge tube to wash the precipitated protein, the tube was centrifuged for 5 min at 1500 × g and the supernatant was decanted; this procedure was repeated five times. The centrifuge tube containing the precipitated protein was then dried in an oven at 75 °C for at least 3 h. A 3 mg sample of dried protein was weighed into a tin capsule. For reference, 2 μL (for honey samples) or 1 μL (for protein samples) of olive oil was pipetted into a tin capsule for use as the standard control sample.

All δ^13^C values are related to the Pee Dee Belemnite (PDB) carbonate standard as follows: δ^13^C(‰) = [(R_s_/R_ref_) − 1] × 1000 where R_s_ and R_ref_ are the ^13^C/^12^C isotopic ratios of the sample and the analytical reference standard respectively.

Calculate apparent C-4 sugar content as follows:

$$C-4\;\text{sugar},\%=\frac{\delta^{13}\;C_{P}-\delta^{13}\;C_{H}}{\delta^{13}\;C_{P}-(-9.7)}\times 100$$

where δ^13^C_P_ and δ^13^C_H_ are δ^13^C values, ‰, for protein and honey, respectively, and -9.7 is the average δ^13^C value for corn syrup, ‰. Report negative values from this calculation as 0%. Product is considered to contain significant C-4 sugar (primarily corn or cane) only at or above a value of 7%.

## Results

### Binding activities of PoAbs

The results estimated by using an ELISA method in Table 1 illustrate the binding activities of both antibodies, SP-1 and SP-2, respectively. The OD values of both antibody at 80000-fold dilution were 6.73 and 7.71 times that of the unimmunized rabbit serum (OD at 1000-fold dilution), respectively. This suggests that both antibodies possess very high binding activities to MRJP1, indicating they met the standard of titer for immunological experiments.

**Table 1:** The titer of the PoAbs.

PoAb	Dilution ratio	A_450_-A_630_ value	P/N value*
**SP-1**	1250	1.5263±0.1197^a^*	14.09
	2500	1.4983±0.3085^a^^b^	13.83
	5000	1.255±0.0862^a^^b^^c^	11.59
	10000	1.1047±0.0647^b^^c^^d^	10.20
	20000	1.021±0.1026^c^^d^	9.43
	40000	0.8773±0.0702^c^^d^	8.10
	80000	0.7293±0.1401^d^	6.73
	Negative control	0.1083±0.0285^e^	-
**SP-2**	1250	1.424±0.2894^a^	20.73
	2500	1.387±0.0874^a^	20.19
	5000	1.381±0.3611^a^	20.10
	10000	1.144±0.0674^a^^b^	16.65
	20000	1.059±0.4256^a^^b^	15.41
	40000	0.887±0.3258^a^^b^	12.91
	80000	0.530±0.0845^b^^c^	7.71
	Negative control	0.069±0.0330^c^	-

*P/N value, the positive/negative value, which was the ratio of the optical density value of the testing sample to the negative control sample. Values were calculated according to the formula P/N > 2.1. (+) means positive and (-) means negative. (*p*<0.01)

^a~e^ the significance of the difference between the data, the significant level α = 0.05. The numbers to be compared have the same letter as the difference is not significant, and the data without the same letter is considered to be significantly different.

### Standardization of ELISA with both antibodies

**Fig 1:**
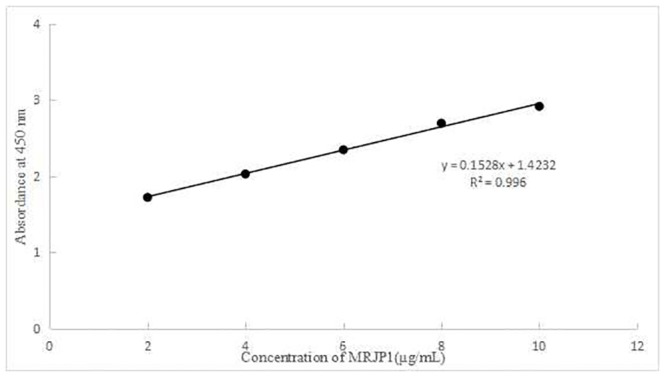
Standard curve of the MRJP1.

Based on ELISA with both antibodies, we generated the calibration curves: y = 0.1528x+1.4232 (R^2^ = 0.996) for MRJP1 (Fig 1) in a concentration ranging from 2 to 10 μg /mL of MRJP1 in PBS.

### Measurement of colloidal gold particles

**Fig 2:**
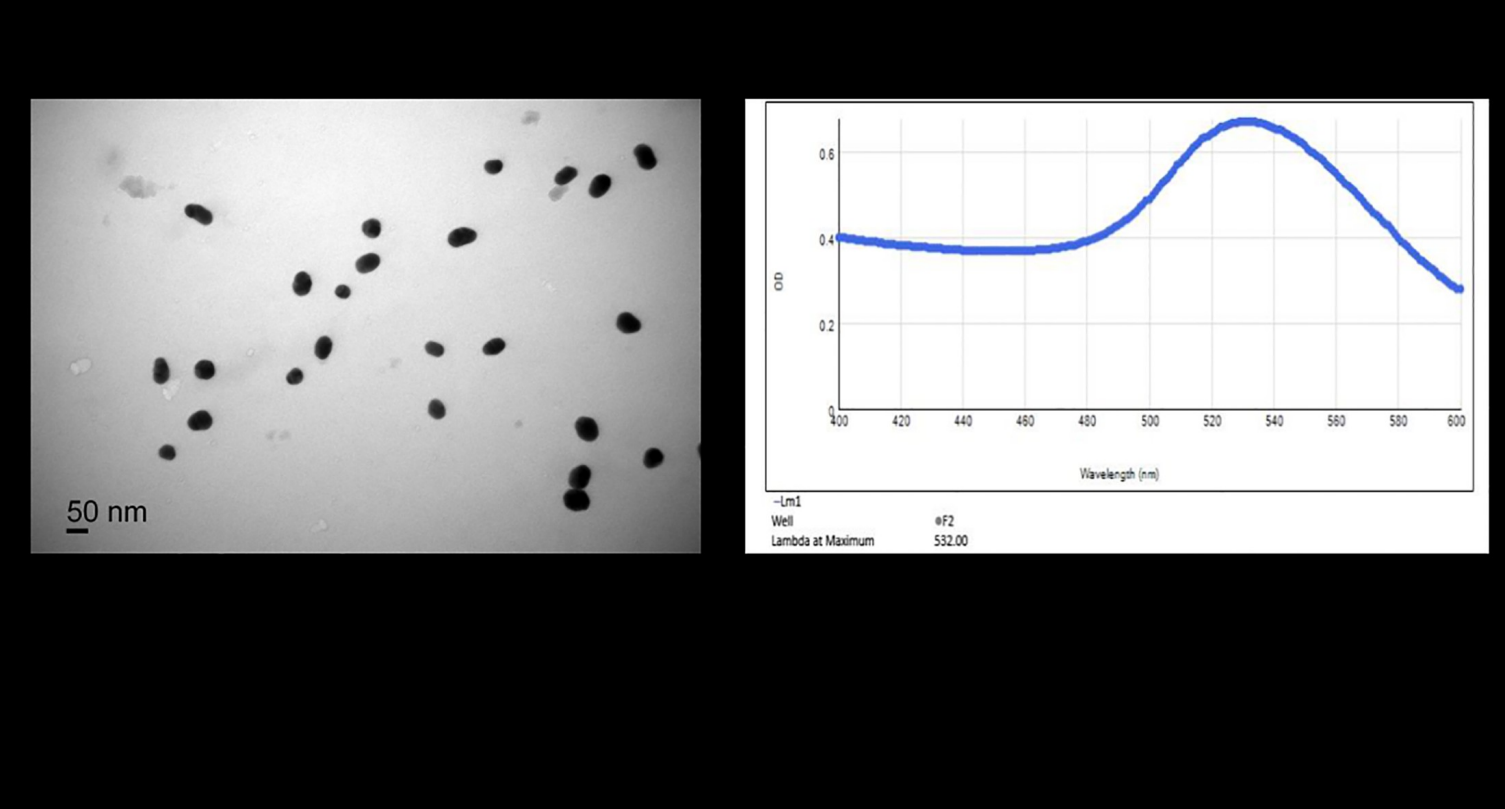
Transmission electron micrograph(A) and visible spectrum (B) of the colloidal gold.

In this experiment, transmission electron microscopy showed that the diameter of the colloidal gold particles was 35–45 nm (Fig 2A). The visible spectrum (Fig 2B) showed that it had the maximum at 532 nm. The absorbance gives the colloidal gold solution an average particle size distribution of about 41 nm. Although the particle size distribution and peak width observed during transmission electron microscopy were not particularly satisfactory, it was combined with the optimal amount of PoAbs, the colloidal gold was stable. It was demonstrated that the colloidal gold particles in this experiment met the requirements of 40 nm colloidal gold experiment.

### Optimization of pH and PoAb concentration

**Fig 3:**
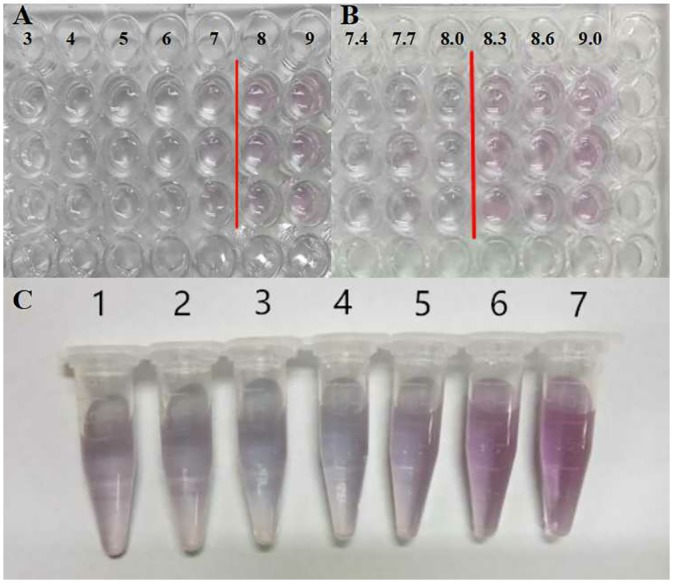
Optimal conditions. **(A)** Optimization of pH value in conjunction with colloidal gold. pH values from left to right are 3, 4, 5, 6, 7, 8, 9, respectively; **(B)** pH values from left to right are 7.4, 7.7, 8, 8.3, 8.6, 9.0, respectively; **(C)**The Optimization amount of SP-1 in conjunction with colloidal gold.

The optimization of pH value to detect the presence of MRJP1 is the lowest pH value of the well that stays red. The primary measurement results (Fig 3A) of pH, 3, 4, 5, 6, 7, 8, 9 showed the optimization of pH value in conjunction with colloidal gold was (pH)X = 8. In accordance with this measurement, the range of pH from 8 to 9 was considered the optimum value based on the initial determination. Further measurement results under the pH gradients, 7.4, 7.7, 8, 8.3, 8.6, 9.0 showed that the pH values staying red were 8.3, 8.6 and 9.0 (Fig 3B). Finally, pH 8.6 was considered as the best pH value for the protein is positively charged and is more stable for the binding, because the pH value of the colloidal gold should be slightly higher than the pI of the actual colloidal gold probe in preparation.

In order to determine the optimal amount of PoAb SP-1 used in conjunction with colloidal gold, seven different concentrations of SP-1 solutions were made by adding 0 to 6 μL of SP-1 (1 mg/mL) solution to 1 mL colloidal gold solution (pH = 8.6) separately (Fig 3C). It was shown that the colloidal gold in the tube without protein and protein deficiency is unstable, which resulted in poly sink phenomenon and color changing from red to purple. When the SP-1 content reaches or exceeds the minimum stable quantity, the colloidal gold becomes stable and the red color remains unchanged. We found that the solution color in tube 5 was the closest to the color of the standard colloidal gold solution. Also in general, the amount of protein should be slightly more than that of the amount in conjunction with the colloidal gold. Thus, the optimized amount of SP-1 is 5 μg.

### The lowest detection limit and the specificity of GSIS

**Fig 4:**
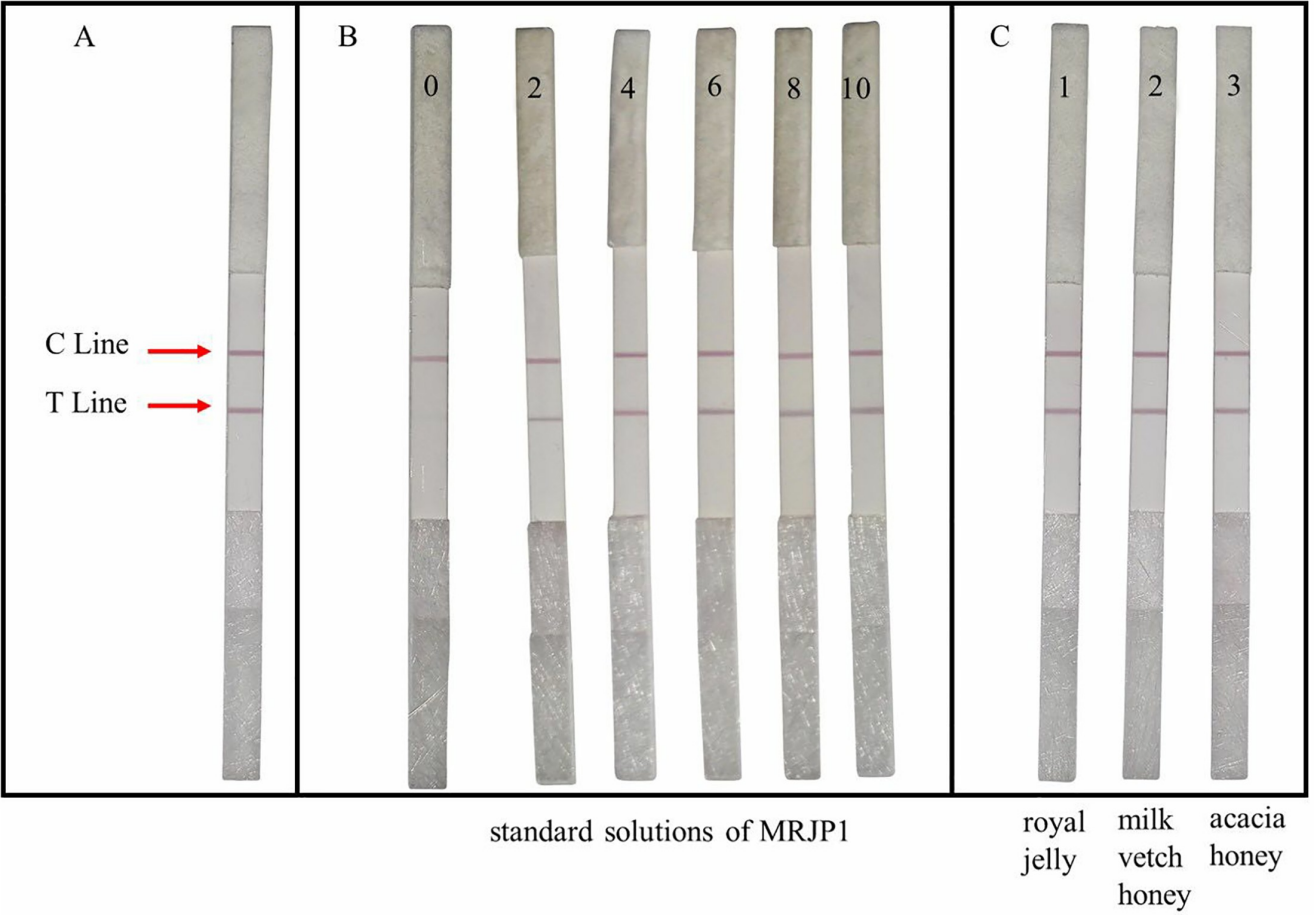
The lowest detection limit and the specificity of IC strip test. **(A)** Performance tests of the colloidal gold immunochromatographic strips; **(B)** Standard solutions of MRJP1 at each concentration of 0, 2, 4, 6, 8 and 10 μg/mL were tested; **(C)**The detection of MRJP1 by using the immunochromatographic strip. **(1)** royal jelly; **(2)** milk vetch honey; **(3)** acacia honey.

Fig 4A showed that C line appeared on the strip testing MRJP1 protein standard solution, indicating the strips were effective. Then, a series of MRJP1 standards (2, 4, 6, 8, 10 μg/mL in 0.01 M PBS) and a blank control with 0.01 M PBS were tested by using IS. Fig 4B showed that the colors of the C line and the T line were illegible when the MRJP1 concentration of test solution was below 2 μg/mL, but legible with increasing of MRJP1 concentration from 2 to 10 μg/mL in the test solution. The result suggested that the cut-off value for the strips to detect MRJP1 is 2 μg/mL.

#### Detection of MRJP1 contents in pure honey and honey adulteration by ELISA

The data in Table 2 showed that the MRJP1 contents of different honey samples obtained by ELISA. The MRJP1 contents of pure milk vetch honey and acacia honey contained was 1.32±0.11 mg/g. The MRJP1 contents in various honey adulteration with rice syrup or corn syrup 70%, 50%,70% and 100% were 0.9, 0.6, 0.4 and 0.02~0.4 mg/g, respectively.

**Table 2:** The MRJP1 contents in pure honey and honey adulteration by ELISA.

No.	Milk vetch honey/%	Rice syrup/%	Corn syrup/%	Portion of adulteration/%	The content of MRJP1 (mg/g)
1	100			0	1.32±0.11
2	70	30		30	0.91±0.17
3	50	50		50	0.64±0.06
4	30	70		70	0.42±0.11
5		100		100	0.04±0.04
6	70		30	30	0.92±0.05
7	50		50	50	0.63±0.04
8	30		70	70	0.40±0.06
9			100	100	0.02±0.03

### Sample analysis of the strip

Fig 4C showed the detection results for the strip of different test samples. The C line appeared on all strips contacted RJ, milk vetch honey and acacia honey, respectively, indicating the validity of the strip. The appearance of T line on strips clearly demonstrated that the test sample contains MRJP1.

### Detection of honey adulteration with GSIS

**Fig 5:**
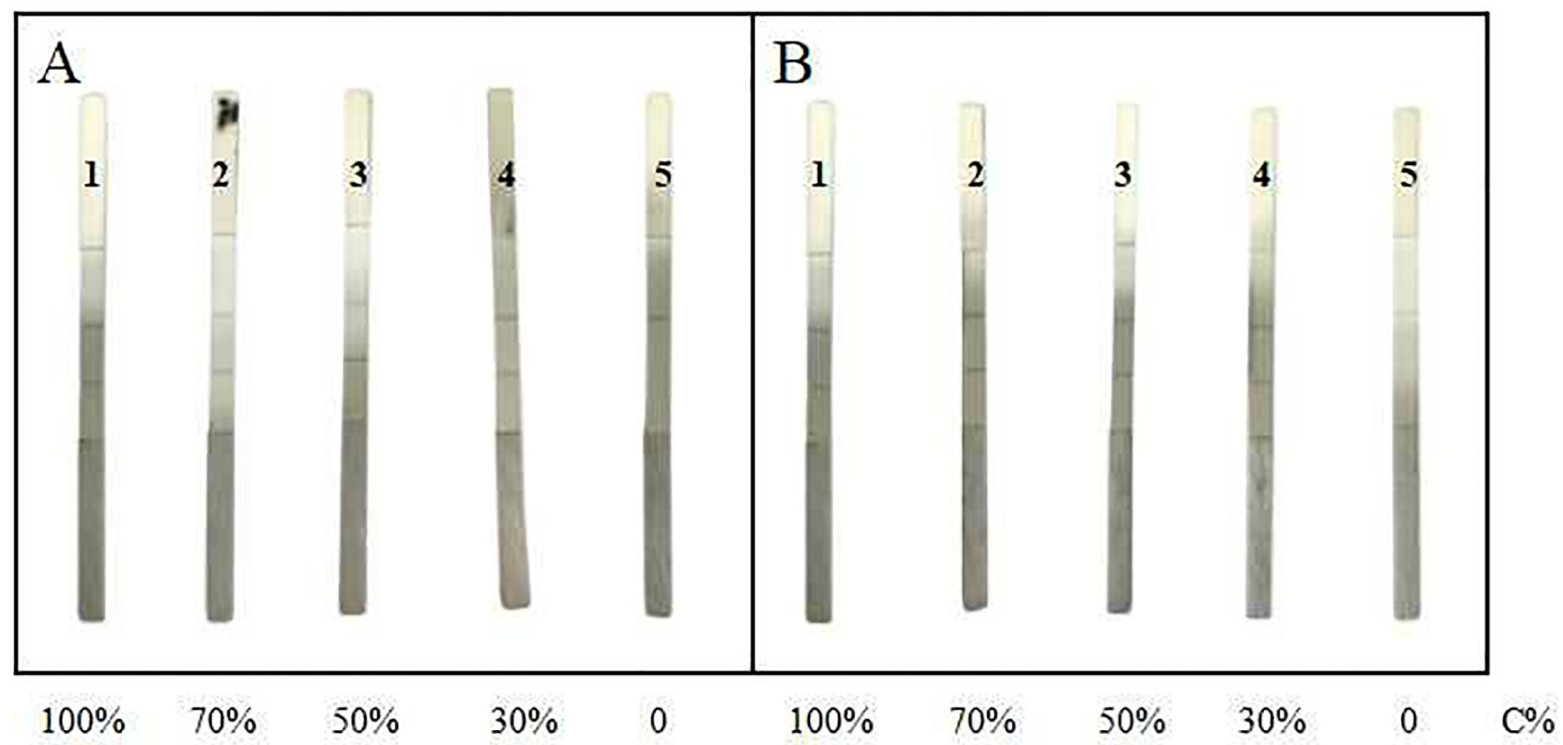
The detection of adulterated milk vetch honey by using the immunochromatographic strip. **A: (1)** milk vetch honey (honey concentration 100%); **(2)**milk vetch honey: corn syrup = 7:3 (honey concentration 70%); **(3)** milk vetch honey: corn syrup = 5:5 (honey concentration 50%); **(4)**milk vetch honey:corn syrup = 3:7 (honey concentration 30%); **(5)** corn syrup (honey concentration 0); **B: (1)** milk vetch honey (honey concentration 100%); **(2)** milk vetch honey: rice syrup = 7:3 (honey concentration 70%); **(3)** milk vetch honey: rice syrup = 5:5 (honey concentration 50%); **(4)**milk vetch honey: rice syrup = 3:7 (honey concentration 30%); **(5)**rice syrup (honey concentration 0).

The test results of the milk vetch honey adulteration experiment were shown in Fig 5. All strips treated with honey adulteration samples containing corn syrup [Fig 5A(1)–5A(4)] and rice syrup [Fig 5B(1)–5B(4)] showed positive results. However, the colors of both C line and T line were stable when the honey concentration in test solution was higher than 30%, or the MRJP1 content in solution was more than 0.4 mg/g (Table 2), the T line becomes illegible when the honey concentration was lower than 30%, suggesting that high honey concentration strongly influenced the movement of gold-labeled antibody on NC membrane and lead to a false positive.

### Detection of honey adulteration by EA-IRMS

To validate the sensitivity and reliability of GSIS test set by us, pure natural milk vetch honey, the same honey adulteration samples containing corn syrup and rice syrup used as above was detected by EA-IRMS. The judged results according to C-4 sugar/% (Table 3) showed the same information as GSIS tests. The C-4 sugar contents of samples containing more than 30% rice syrup and corn syrup were higher than 11.53% and 32.33%, respectively, and the value above 7% was judged to be adulterated honey. The EA-IRMS test results are basically matched with the GSIS test results. However, when there is only pure syrup, such as rice or corn syrup of the sample in this experiment, EA-IRMS can not show whether the sample contains C-4 sugar, that is, it is not intuitive to judge whether the sample is adulterated or fake honey. There are limitations compared to the strip detection method.

**Table 3:** Detection of pure honey and honey adulteration by EA-IRMS.

No.	Milk vetch honey/%	Rice syrup/%	Corn syrup/%	Portion of adulteration/%	δ^13^C_H_	δ^13^C_P_	C-4 sugar/%**	Indication of adulteration
1	100			0	-23.57	-24.33	5.19	No
2	70	30		30	-24.20	-26.09	11.53	Yes
3	50	50		50	-24.98	-27.15	12.44	Yes
4	30	70		70	-25.72	-28.39	14.29	Yes
5		100		100	-26.52	/^a^	/	/
6	70		30	30	-19.63	-24.35	32.33	Yes
7	50		50	50	-19.51	-24.46	33.54	Yes
8	30		70	70	-15.20	-23.62	60.49	Yes
9			100	100	-10.44	/	/	/

^a^ No protein, indicating adulteration.

**C-4 sugar/%, the C-4 sugar/% of honey sample above 7% is judged as adulteration [32].

## Discussion

The assay, lateral-flow immunochromatography strip methods were established by using two PoAbs, SP-1 and SP-2 on the basis of ELISA detection. The method could detect MRJP1 in honey samples. The best of pH value (pH 8.6) and PoAb SP-1 amount (5 μg/mL) in conjunction with colloidal were determined, respectively. The color test of the MRJP1 standard sample was sensitive and the cut-off value (sensitivity) of GSIS in detecting MRJP1 is 2.0 μg/mL in a solution. MRJP1 could be detected in the pre-treated honey samples by the GSIS. Validation analysis with RJ, milk vetch honey, acacia honey and honey adulteration containing rice syrup and corn syrup with different ratios demonstrated that the GSIS could show consistent T line when the test samples contain crude RJ or more than 30% pure honey. The detection of adulterated honey by sandwich ELISA and EA-IRMS method showed that the MRJP1 content was basically consistent with that of the GSIS. The ELISA method takes a long time to measure, and the EA-IRMS method cannot determine whether the pure syrup sample is a qualified honey.

The successful utilization of the specific-polyclonal antibodies in our present study laid the foundation for the subsequent preparation of more specific GSIS with monoclonal antibodies in the future. Furthermore, the GSIS based on specific-polyclonal antibodies to detect honey adulteration is highly sensitive.

Moreover,our results of GSIS tests on honey adulteration were verified by EA-IRMS. The EA-IRMS method developed by JW White [34] is an effective tool for judging adulterated honey. The qualified honeys judged with the EA-IRMS method by Qinhuangdao Entry–Exit Inspection and Quarantine Bureau in China were exported to the USA, Canada, Germany, Spain, Belgium, Japan, etc. and gained smooth customs clearance in these countries, and customers did not raise any concerns, which proved that the method is reliable [33].

However, the GSIS possesses its weaknesses because it may cause interference to the qualitative judgment of color-distinguishing results. Therefore, semi-quantitative and quantitative detection methods to improve the accuracy of test strip inspection will be explored in the follow-up study. In addition, the process of pretreatment of honey required extraction and dialysis is time-consuming. Thus, it is necessary to explore a more straightforward method for protein separation and purification in future studies.

In summary, as this method has several advantages: low cost, fast detection, easy to use, not need high-cost equipment, it is great potential for high throughput assay. We believe that the test strip test method of this paper may provide a new tool for existing honey adulteration detection technology.

## Supporting information

S1 Table Supplementary data for the titer of the PoAbs. (XLSX)

S1 File Immunoassay strip for honey adulteration detection. This is a picture for the detection of honey adulteration by the immunoassay strips studied in this paper.
(PDF)

## Abbreviations

BSA: bovine serum albumin
C line: Control line
CB: carbonatebicarbonate buffer
CGIS: colloidal gold immunochromatographic strip
EA-IRMS: isotope ratio mass spectrometry
ELISA: enzyme-linked immunosorbent assay
GNPs: colloidal gold nanoparticles
GSIS: gold sandwich immunochromatographic strip
HRP: horseradish peroxides
IS: immunochromatographic Strip
MRJPs: major RJ proteins
OD: optical density
PBS: phosphate buffered saline
PBST: PBS with 0.05% (v/v) Tween-20
PDB: Pee Dee Belemnite
PEG 2000: polyethylene glycol 2000
PI: protein isoelectric point
PoAbs: polyclonal antibodies
PVA: polyvinyl alcohol
PVC: polyvinyl chloride
PVP: polyvinyl pyrrolidone
RJ: royal jelly
T line: test line
TMB: 3, 3′, 5, 5′-tetramethylbenzidine
