# Supplementary figures and images for: Novel polyclonal antibody-based rapid gold sandwich immunochromatographic strip for detecting the major royal jelly protein 1 (MRJP1) in honey
Source: PLoS One. 2019 Feb 19;14(2):e0212335. doi: 10.1371/journal.pone.0212335 (PMC6380560; doi:10.1371/journal.pone.0212335)

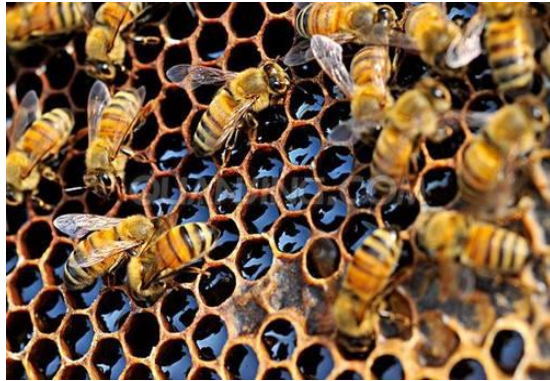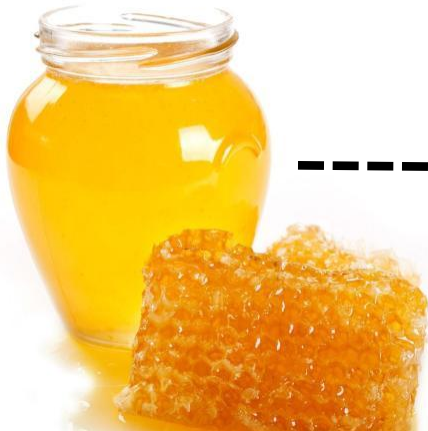

Control line  
Test line

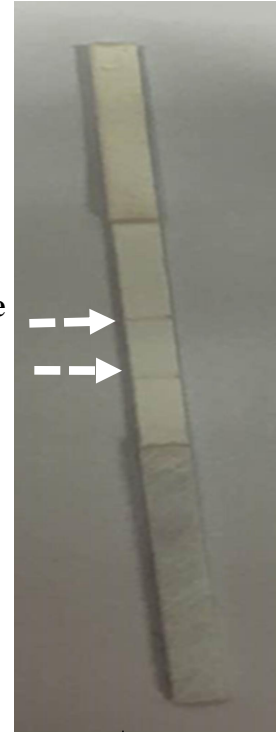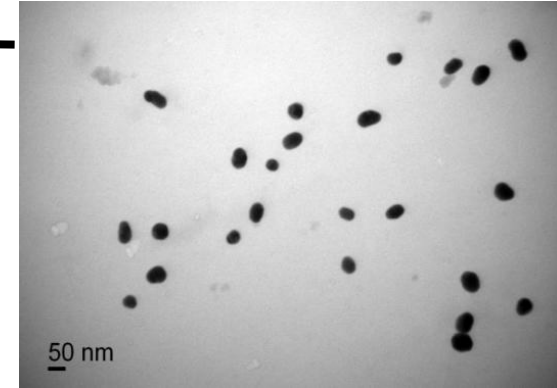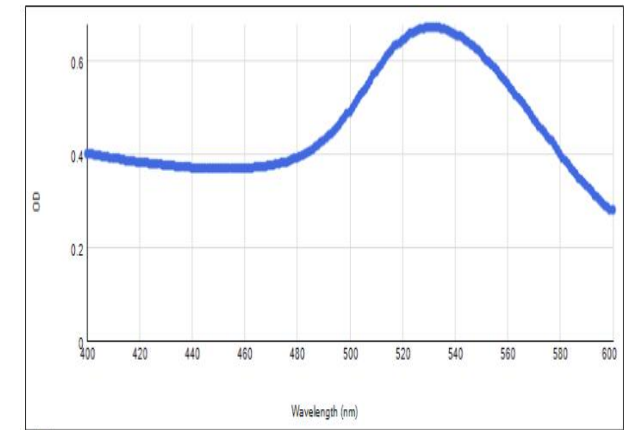

-Ln1  
Well #F2  
Lambda at Maximum 532.00

Supplement: S1 File — This is a picture for the detection of honey adulteration by the immunoassay strips studied in this paper. (PDF) [file pone.0212335.s002.pdf]
